# The antibacterial activity and toxin production control of bee venom in mouse MRSA pneumonia model

**DOI:** 10.1186/s12906-020-02991-8

**Published:** 2020-07-27

**Authors:** Ryong Kong, Young-Seob Lee, Dam-Hee Kang, Shu Wang, Qianqian Li, Dong-Yeul Kwon, Ok-Hwa Kang

**Affiliations:** 1 Department of Oriental Pharmacy, College of Pharmacy and Wonkwang-Oriental Medicines Research Institute, Wonkwang University, Iksan, Jeonbuk 54538 Republic of Korea; 2 Department of Herbal Crop Research, National Institute of Horticultural & Herbal Science, RDA, 92 Bisanro, Eumsung, Chungbuk 27709 Republic of Korea

**Keywords:** MRSA, Bee venom, Toxin, Pneumonia

## Abstract

**Background:**

The current antimicrobial therapy is still important for the treatment of pneumonia due to MRSA infection, but there are some limitations, including the route of administration, side effect profile, and increased microbial resistance patterns. Therefore, we investigated whether BV, which shows a strong antimicrobial effect against MRSA, would be effective in a pneumonia model.

**Methods:**

In vitro, we checked MIC, qRT-PCR, western blot, ELISA, LDH-assay. In vivo, we checked survival rate, gross pathological change, histopathology, lung bacterial clearance assay, and the expression of inflammatory related gene.

**Results:**

The minimum inhibitory concentration of BV against MRSA is 15.6 μg/ml by broth dilution method. The production of toxins and related gene were reduced by BV in MRSA. The secretion of cytokines were decreased by treatment with BV in 264.7 RAW macrophages stimulated by MRSA Also, BV protected A549 from pathogenicity of MRSA. Bee venom reduced the number of bacteria in the lungs and alleviated the symptoms of MRSA-induced pneumonia in mouse.

**Conclusion:**

BV inhibited the virulence of the bacterium and the number of bacterial cells present in lung tissue, thereby alleviating the symptoms of pneumonia in mice. This study suggested that BV may be a candidate substance for the treatment of pneumonia caused by MRSA infection.

## Background

MRSA is a common cause of nosocomial pneumonia and healthcare-associated pneumonia [1]. The main cause of MRSA-induced pneumonia is toxin. The α-hemolysin, a member of the β-barrel pore-forming toxin family, is the major virulence factor secreted by strains of *S. aureus* [2]. The α-hemolysin is necessary for the pathogenesis of staphylococcal pneumonia. Bubeck Wardenburg et al. have reported that *S. aureus* mutants lacking *hla* (the gene encoding α-hemolysin) cannot cause neutrophil-mediated inflammatory lung injury in pneumonia model [3]. Similar to most staphylococcal extracellular proteins, the α-toxin is not expressed constitutively but is tightly controlled by an array of extracellular and intracellular signals. At least three global regulatory loci the accessory gene regulator (*agr*), the staphylococcal accessory gene regulator (*sarA*), and the staphylococcal accessory protein effector (*sae*) appear to coordinately control the *hla* expression in *S. aureus* in vitro. In addition, two *sarA* homologs, *rot* and *sarT*, repress the *hla* expression. The *agr* locus affects a direct positive impact on *hla* expression, whereas sarA positively affects the *hla* expression via both *agr*-dependent and *agr*-independent pathways. The *sae* locus consists of a two-component signal transduction system, encoded by *saeS* (sensor) and *saeR* (response regulator), which positively controls the expression of *hla* at the transcriptional level. In addition, the complex transcriptional profile of sae activation is influenced by *agr*, as well as by certain environmental stimuli, and may also be influenced by sigma factor B (*sigB*) [4].

The current antimicrobial therapy is still important for the treatment of pneumonia due to MRSA infection, but there are some limitations, including the route of administration, side effect profile, and most importantly, increased microbial resistance patterns [5]. Therefore, it is necessary to search materials that overcome the obstacles to the treatment of pneumonia caused by MRSA. Some researchers have already reported on the studies that is treatment of pneumonia by using natural product or peptide. Bae et al., reported that SAL200, which is peptidoglycan-degrading enzyme, reduced the pulmonary bacterial burden in MRSA induced pneumonia [6]. Xia et al., reported that the combination of LysGH15 (peptide) and apigenin (natural product) shows therapeutic potential for treating MRSA induced pneumonia [7]. Also, several researchers demonstrated that natural products target virulence and exhibit therapeutic effect against MRSA-induced pneumonia [8, 9]. We have also performed the study on treatment of pneumonia by using material used in traditional Eastern medicine.

Bee venom (BV) has been used in traditional Eastern medicine to reduce pain and treat chronic inflammatory diseases. Various studies have demonstrated the biological activity of BV. In addition, BV has been reported to have various physiological activities such as antibacterial, anticancer, and anti-inflammatory effects [10–12]. BV contains various peptides, amines, nonpeptide components, and free amino acids, which are presumed to have anti-inflammatory, analgesic, and anticancer effects. BV contains physiologically active peptides such as melittin, secapin, mast cell degranulating (MCD) peptide, apamin, etc. [13]. Melittin is a major component of the honey bee (*Apis mellifera* L.) venom and an amphiphilic peptide that has 26 amino acid residues. Melittin has been used in tumor-bearing mouse research because of its cytotoxicity to tumor cells and capacity to inhibit cell growth or induce apoptosis and necrosis [14]. Lee et al. have reported the therapeutic effects of melittin and its detailed mechanisms of action against several inflammatory diseases, including skin, nerve, liver, atherosclerosis, and arthritis [15]. Secapin is a polycationic peptide with 25 amino acid residues, which contains an intramolecular disulfide bridge; it represents approximately 0.5% of the dry weight of honeybee venom [16]. Lee et al. have demonstrated that secapin functions as an antifibrinolytic, anti-elastolytic, and antimicrobial agent in a manner similar to that of serine protease inhibitors [17]. In addition, BV has been reported to show an antibacterial effect on MRSA [18]. However, no pneumonia mouse model of MRSA has been reported. Therefore, we investigated whether BV, which shows a strong antimicrobial effect against MRSA, would be effective in a pneumonia model.

## Methods

### Bacterial strains and reagents

**Fig. 1:**
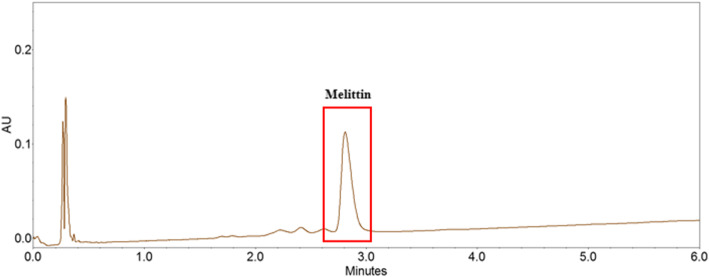
Measurement of melittin content in bee venom using UPLC analysis

ATCC 33591 (reference strain, HA-MRSA) and USA300 (wild-type LAC strain, CA-MRSA) were used in this study. ATCC 33591 was incubated in Mueller–Hinton broth (MHB), and USA300 was incubated in tryptic soy broth (TSB) at 37 °C for 24 h. Bee venom (BV) which is collected by bee venom collector apparatus (Chunglin Biotech, Ansan, Korea) to natural honeybees (*Apis mellifera* L.) was purchased from Korea Purified bee venom agricultural association corporation (Changnyeong, korea). The contents of melittin, the main component of bee venom, were analyzed by UPLC (Agilent 1290, USA) according to pH and temperature changes. The analysis conditions are shown in Table 1, and the results of the UPLC analysis are shown in Fig. 1. The content of melittin in bee venom was 60.2%.

**Table 1:** UPLC conditions for melittin analysis

Item	Condition		
Column	Halo ES-C18(2.1 × 100 mm, 2.7 μm, Advanced materials technology, USA)		
Flow rate	0.8 mL/min		
Column temperature	50 °C		
Injection volume	2 μl		
Wavelength	220 nm		
Mobile phase	**Time (min)**	**20 mM TFA**^**a**^**/MeCN(%)**	**20 mM TFA/H**_**2**_**O(%)**
	0	40	60
	6	45	55

^a^*TFA* trifluoroacetic acid

### Mouse model of intranasal lung infection

Balb/c mice (7 weeks old, females) were purchased from Orient Bio (Seongnam, Korea). *S. aureus* USA300 was grown in TSB at 37 °C to an optical density at 600 nm (OD_600_) of 0.6; 50 mL of the culture was centrifuged (3000×g, room temperature, 5 min), and the pellet was resuspended in 1.2 mL of sterile phosphate-buffered saline (PBS, pH 7.4). For both lethality and histopathology assays, the mice were randomly divided into seven groups (normal, PBS, control, BV 0.125, BV 0.25, BV 0.5 and LZD 10 mg/kg/day groups; *n* = 5 each) after intranasal infection with 30 μL of an *S. aureus* suspension [1 × 10^10^ colony-forming units (CFU) per 30 μL]. After 30 min, BV (0.125, 0.25, and 0.5 mg/kg/day) and LZD was administered by intraperitoneal injection. The control group mice were treated with 100 μL of sterile PBS on the same schedule as a solvent control. For histopathological analysis, the mice were anesthetized and euthanized by cervical dislocation 24 h post-infection. Ketamine (10 mg / mL) / Xylazine (10 mg / mL) was used for mouse anesthesia and administered 10 μL / g by intraperitoneal injection The lungs were fixed in 10% formaldehyde, stained with hematoxylin and eosin (H&E), and examined by light microscopy.

### The measurement of minimum inhibitory concentration (MIC)

The minimum inhibitory concentration (MIC) was determined using a broth microdilution method according to the Clinical and Laboratory Standards Institute guidelines [19]. Serial, two-fold dilutions of BV and 10 μL of antibiotics were prepared in 100 μL of MHB and TSB in 96-well microplates. The microplates were inoculated with MRSA adjusted to 0.5 McFarland standard (approximately 10 μL of 1.5 × 10^8^ CFU/mL; final density 1.5 × 10^6^ CFU/mL). MIC was determined as the lowest BV concentration suppressing the growth of MRSA after 24 h of incubation at 37 °C.

### RNA extraction and quantitative reverse transcription polymerase chain reaction (qRT-PCR)

Total RNA was isolated from ATCC 33591 using an E.Z.N.A.® bacterial RNA kit (Omega Bio-tek, Norcross, GA, USA) according to the manufacturer’s instructions. Total RNA was also isolated from homogenized lung tissue using an easy-BLUE™ total RNA extraction kit (iNtRon Biotechnology, Inc., Seongnam, Korea). Total RNA was dissolved in diethyl pyrocarbonate-treated distilled water. A spectrophotometer (BioTek, Winooski, VT, USA) was used to evaluate RNA purity by measuring the absorbance ratio at 260 and 280 nm. Complementary DNA was synthesized using the QuantiTect reverse transcription kit (Qiagen, Seoul, Korea) according to the manufacturer’s instructions. Real-time quantitative reverse transcription–polymerase chain reaction (qRT–PCR) was performed in triplicate using a Power SYBR® Green PCR master mix (Applied Biosystems, Foster City, CA, USA) and a StepOnePlus real-time PCR system (Applied Biosystems). The amplification conditions were 95 °C for 10 min, followed by 40 cycles of 95 °C for 15 s and 60 °C for 1 min. Expression levels of target genes relative to the endogenous reference gene, β-actin, were calculated by the delta–delta cycle threshold method using the StepOne software v2.3 (Applied Biosystems). The primer sequences are listed in Table 2.

**Table 2:** Sequences of oligonucleotide primers designed for real-time PCR

Gene	Primer sequence
*Saa3*	
Forward	GCCTGGGCTGCTAAAGTCAT
Reverse	TGCTCCATGTCCCGTGAAC
*Orm1*	
Forward	GTGTGTCTATAACTCCACCCATC
Reverse	CCCATGTTTCCTCAGCACTAT
*CXCL9*	
Forward	GGCACGATCCACTACAAATCC
Reverse	GGTTTGATCTCCGTTCTTCAGT
*Pon1*	
Forward	GGACTAACTTTCTTTAGCATGGGC
Reverse	TTCTAACTCTGACACTGCTGGCTCC
*GAPDH*	
Forward	TGGGCTACACTGAGCACCAG
Reverse	GGGTGTCGCTGTTGAAGTCA
*hla*	
Forward	TTGGTGCAAATGTTTC
Reverse	TCACTTTCCAGCCTACT
*agrA*	
Forward	TGATAATCCTTATGAGGTGC
Reverse	CACTGTGACTCGTAACGAAA
*16 s RRNA*	
Forward	CGTGCTACAATGGACAATAC
Reverse	ATCTACGATTACTAGCGATT

### Western blot analysis

MRSA strain ATCC 33591 was grown to an OD_600_ value of 0.9 in MHB with various concentrations of BV for western blotting, and the cells were harvested after 4 h of treatment by centrifugation at 13,000 rpm for 10 min. The harvested bacterial cells were resuspended in the SMART bacterial protein extraction solution (iNtRON Biotechnology) according to the manufacturer’s protocol. Denatured protein lysates (20 μL) were separated by 12% sodium dodecyl sulfate polyacrylamide gel electrophoresis, and the resolved proteins were transferred onto polyvinylidene difluoride membranes. The membranes were incubated with an anti-Staphylococcus alpha hemolysin antibody (diluted 1:500; Abcam, UK) overnight at 4 °C. Goat anti-rabbit IgG (diluted 1:1000; Thermo Scientific, USA) diluted 1:2500 in 5% skim milk was used as a secondary antibody. Immunoreactive protein bands were detected using the EzWestLumi Plus luminol substrate (ATTO Co., Tokyo, Japan) and visualized using an ImageQuant LAS 4000 mini chemical luminescent imager (GE Healthcare Life, Korea).

### Enzyme-linked immunosorbent assay (ELISA)

The MRSA was grown to an OD_600_ value of 0.9 in MHB with various concentrations of BV for ELISA. Preparation of RAW 264.7 cells were prepared in RPMI 1640 (supplementedwith 10% FBS, 100 IU/mL^− 1^ penicillin and streptomycin). Cells were seeded at a density of 10^6^/mL in RPMI, dispensed (100 μL) into 96-well tissue culture plates and then incubated in 5% CO_2_ at 37 °C in an incubator for 18 h to allow adherence. Cell culture media were washed and RPMI 1640 medium (150 μL) was added. *Staphylococcus aureus* supernatants (50 μL) were added to the tissue culture plate. After incubation for 16 h, supernatants were collected, centrifuged (1000 g for 5 min). Cytokines in the supernatant were measured using the OptEIATM human enzyme-linked immunosorbent assay (ELISA; BD Bioscience, San Jose, CA, USA). Each well of the 96-well microplate was coated with capture antibody diluted in coating buffer (0.1 M carbonate, pH 9.5). Each plate was sealed and incubated overnight at 4 °C. After washing three times with phosphate buffered saline (PBS) containing 0.05% Tween 20, nonspecific binding sites were blocked with PBS containing 10% FBS (pH 7.0) for 1 h. One hundred microliters of each sample, TNF-α and IL-6 standards were added to wells and incubated for 2 h at room temperature. One hundred microliters of detection antibody conjugated with and avidin-horseradish peroxidase (HRP) diluted in assay buffer was applied for 1 h. One hundred microliters of substrate solution (tetramethylbenzidine, TMB) was added to each wells and incubated for 30 min at room temperature in the dark. The 50 μL of stop solution (2 M H_2_SO_4_) was added and the absorbance was determined at 450 nm.

### Cell viability and lactate dehydrogenase assays

A549 human lung epithelial cells were cultured in Dulbecco’s modified Eagle’s medium (DMEM) supplemented with 10% heat-inactivated fetal bovine serum (Welgene, Inc., Korea) and 100 μg/mL penicillin. Cell viability was evaluated by an MTS [3-(4,5-dimethylthiazol-2-yl)-5-(3-carboxymethoxyphenyl)-2-(4-sulfophenyl)-2H-tetrazolium] assay (Promega, Madison, WI, USA) following the incubation in the presence of BV for 24 h at the aforementioned temperature in an atmosphere containing 5% CO_2_. The plate was read using an Epoch microplate spectrophotometer (BioTek), and the absorbance was measured at 490 nm. To detect the lactate dehydrogenase (LDH) activity, A549 cells were seeded at 1.5 × 10^4^ cells per well, cultivated for 12 h to attach, and then incubated with a bacterial suspension at 37 °C for 6 h in DMEM without fetal bovine serum (6). Cytotoxicity was determined based on the LDH release using a Pierce LDH cytotoxicity assay kit (Thermo Scientific, USA) according to the manufacturer’s directions and measuring absorbance on a spectrophotometer (BioTek).

### Lung bacterial clearance assay

In the sublethal model, lungs were harvested after the mice were euthanized, and lung tissue was homogenized in 1 mL of sterile PBS. Serial dilutions were prepared in PBS and plated on tryptic soy agar plates. After 24 h of incubation, bacterial colonies were counted, and CFU counts per milliliter of a lung homogenate were calculated.

### Statistical analysis

Data from the experiments are presented as the mean ± SEM. The level of statistical analysis was performed by Scheffe’s test (SPSS. ver.23) for multiple comparisons. *P*-values < 0.05 was considered significant.

## Results

### Minimum inhibitory concentration (MIC) against MRSA

The MIC values of BV and LZD against the two strains, ATCC 33591 and USA300, are presented in Table 3. The growth of *S. aureus* was inhibited at a concentration of 15.6 μg/mL BV. The MIC of BV was almost similar to that of LZD, a strong antibiotic used to treat MRSA. This results showed that BV have antibacterial activity against MRSA.

**Table 3:** The MIC of BV and LZD against MRSA

*S. aureus* strain	MIC (μg/ml)	
	BV	LZD
ATCC33591	15.2 ± 3.2^a^	7.8 ± 1.5
USA300	15 ± 4.4	15.6 ± 2.1

^a^ Mean of triplicate, with standard deviation

### The inhibition effect of BV against toxins production and related gene of MRSA

**Fig. 2:**
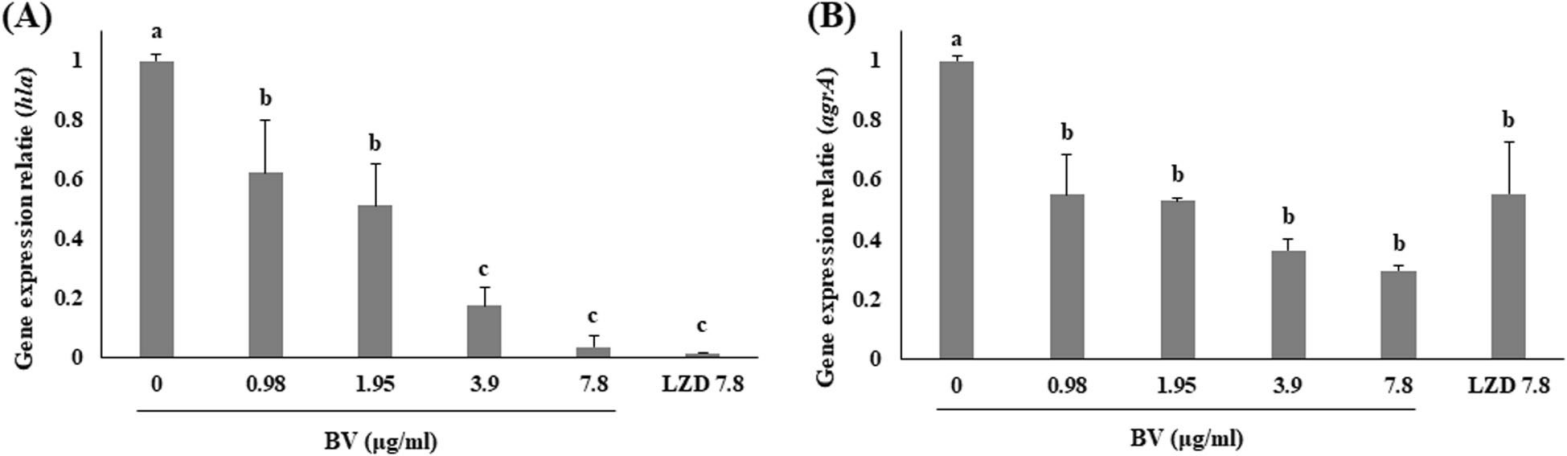
Effects of BV on the mRNA expression of *hla* and *agrA*, as analyzed by qRT–PCR. Cells of ATCC 33591 were treated with serial dilutions of BV (0.98, 1.95, 3.9, and 7.8 μg/mL) and 7.8 μg/mL LZD for 4 h. Different letters (a-d) indicate significant differences at the *p* < 0.05 level, according to analysis of variance followed by Scheffe’s test for multiple comparisons (*p* < 0.05). Values are means ± standard errors

**Fig. 3:**
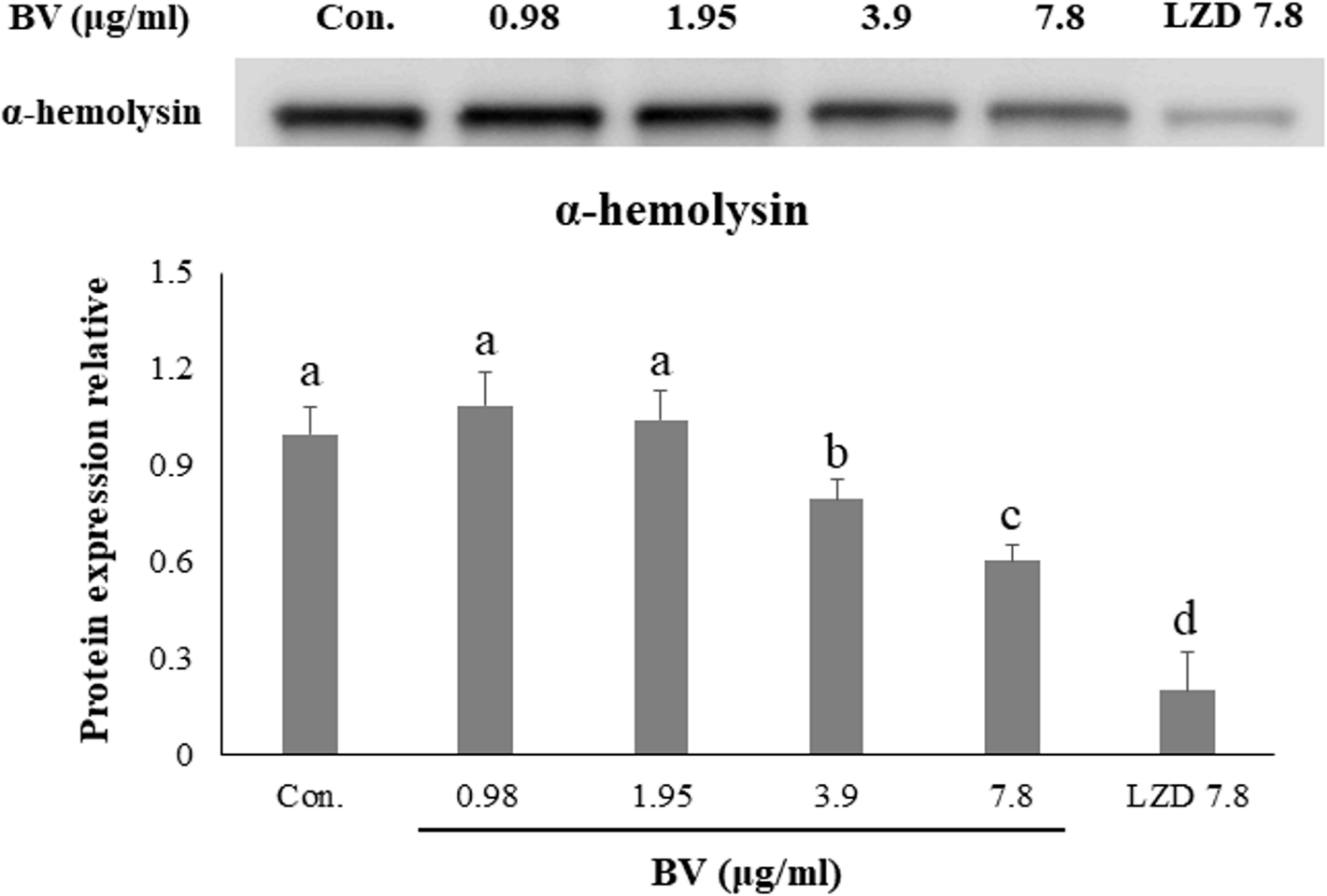
Inhibitory effect of BV on the α-hemolysin production, as analyzed by western blot. Cells of ATCC 33591 were treated with serial dilutions of BV (0.98, 1.95, 3.9, and 7.8 μg/mL) and 7.8 μg/mL LZD for 4 h. Different letters (a-e) indicate significant differences at the *p* < 0.05 level, according to analysis of variance followed by Scheffe’s test for multiple comparisons (*p* < 0.05). Values are means ± standard errors

Expression of the *hla* and *agrA* genes, related to α-hemolysin was confirmed by qRT–PCR. Expression of the four genes was significantly suppressed in ATCC 33591 by the treatment with serial dilutions of BV. In particular, the expression of *agrA* was inhibited when treated with BV than treated with LZD (Fig. 2). Western blotting was used to investigate the effect of BV on toxin production by MRSA. Production of α-hemolysin by ATCC 33591 was significantly decreased by the treatment with serial dilutions of BV. The expression of the toxin showed a similar degree of inhibition when treated with BV compared with LZD treatment (Fig. 3).

### Effects of BV in the pro-inflammatory cytokine release of 264.7 RAW macrophages stimulated by MRSA

**Fig. 4:**
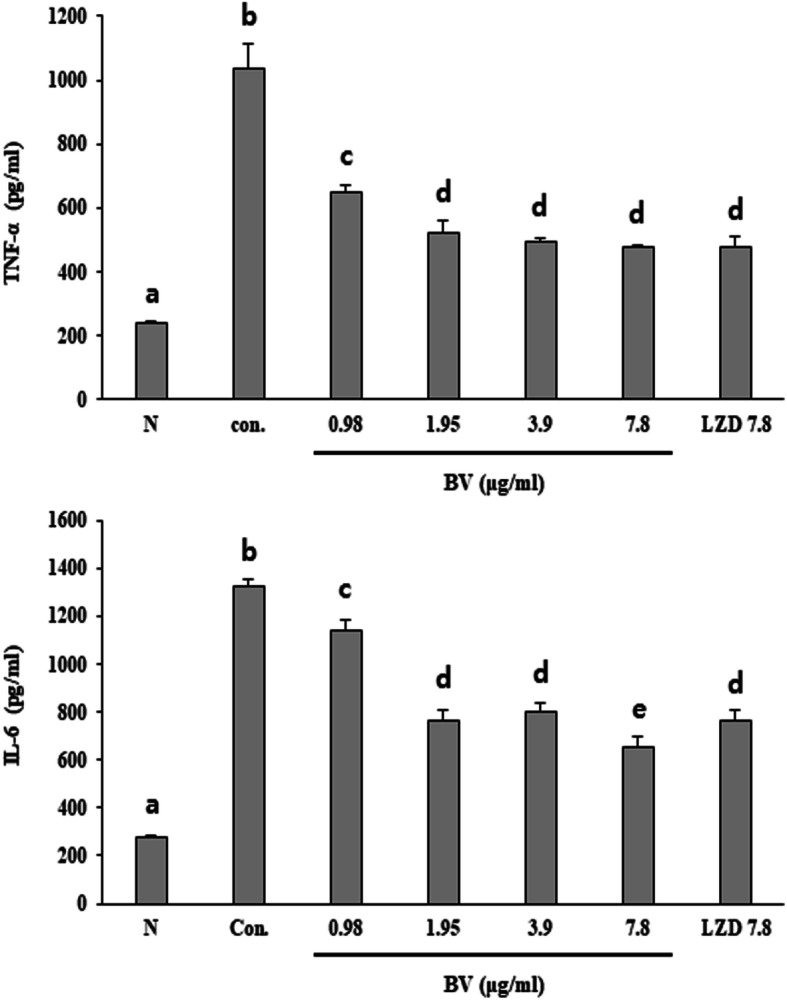
Effect of BV in TNF-α and IL-6 secretion of 264.7 RAW macrophages stimulated by MRSA. After stimulation for 16 h with supernatants of *S. aureus* grown in the presence of graded concentrations of BV in RAW 264.7 cells, TNF-α and IL-6 levels were measured by ELISA. Different letters (a-e) indicate significant differences at the *p* < 0.05 level, according to analysis of variance followed by Scheffe’s test for multiple comparisons (*p* < 0.05). Values are means ± standard errors

ELISA was used to evaluate the effect of BV in tumor necrosis factor (TNF)-α and interleukin (IL)-6 secretion of 264.7 RAW macrophages stimulated by MRSA. The secretion of two cytokines in macrophages significantly increased after stimulation of MRSA. However, the secretion of these cytokines were decreased by treatment with either concentration of BV (Fig. 4).

### Effects of BV on cell viability from virulence of MRSA

**Fig. 5:**
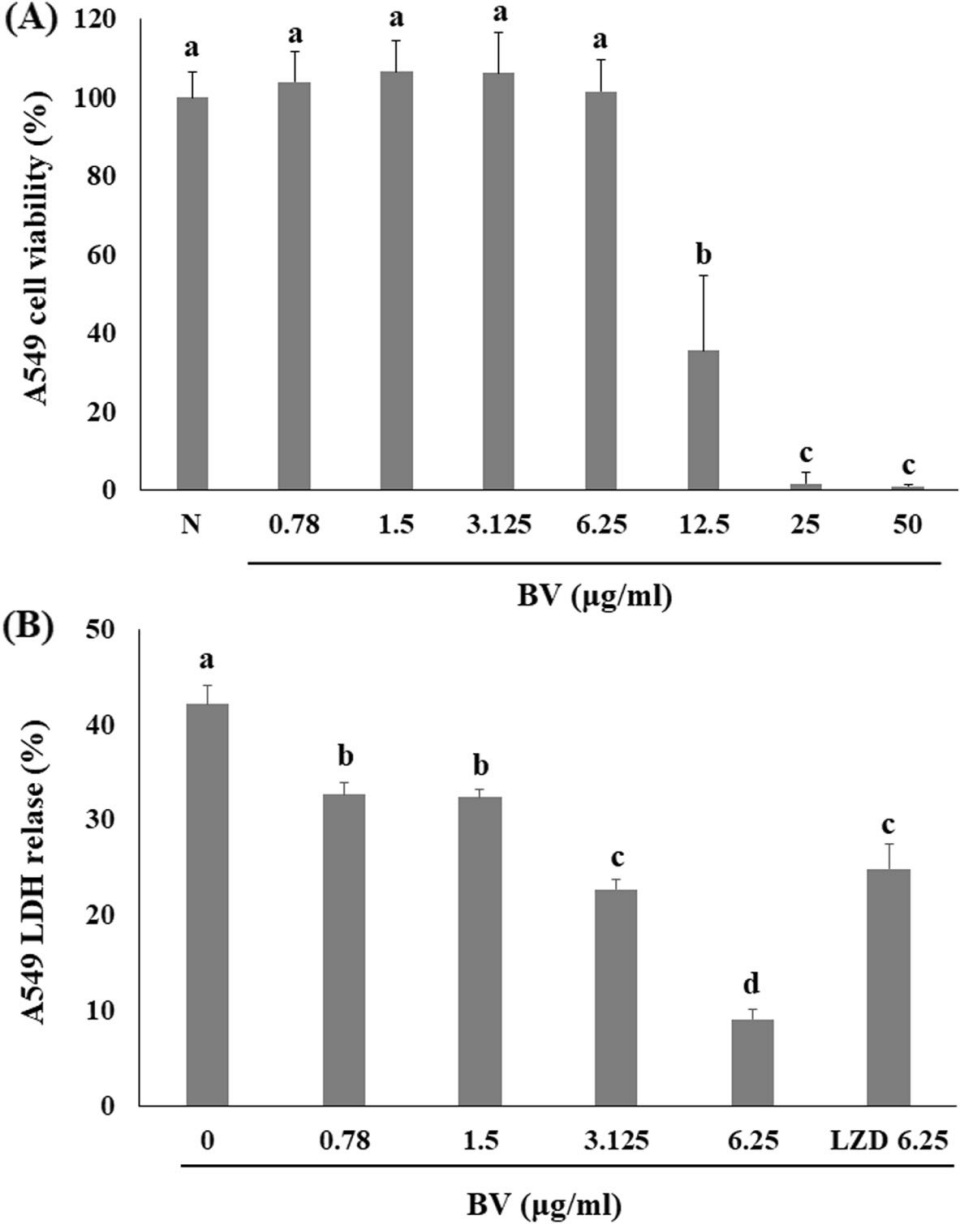
BV attenuates α-toxin-mediated injury of human alveolar epithelial cells. **a** Effects of BV on the viability of A549 cells were evaluated using the MTS assay. **b** The LDH release was determined using A549 cells co-cultured with USA300 and supplemented with the indicated concentrations of BV. Different letters (a-d) indicate significant differences at the *p* < 0.05 level, according to analysis of variance followed by Scheffe’s test for multiple comparisons (*p* < 0.05). Values are means ± standard errors

The MTS assay was performed to measure the viability of A549 human lung epithelial cells treated with BV. BV did not affect the viability of A549 cells at concentrations below 6.25 μg/mL (Fig. 5a). Therefore, the LDH assay was performed to examine whether BV protects cells from MRSA at concentrations below 6.25 μg/mL. As a result, it was demonstrated that the LDH level decreased in a concentration-dependent manner upon BV treatment (Fig. 5b).

### Therapeutic effect of BV against MRSA-induced pneumonia

**Fig. 6:**
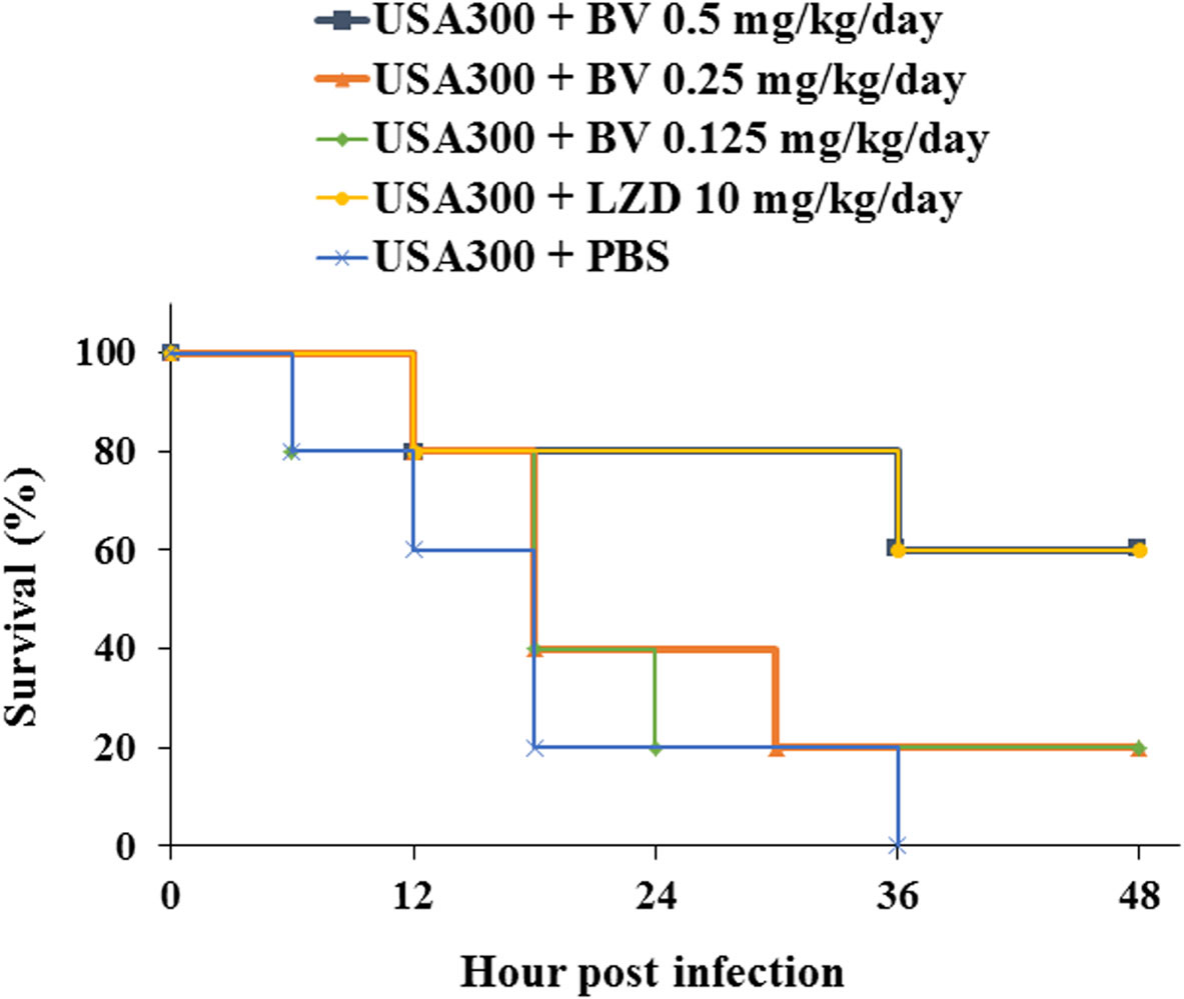
Protection of model mice against MRSA-induced pneumonia by BV. Groups (*n* = 5) of mice were infected with USA300, and survival of the mice was monitored for 48 h after BV and LZD treatment

**Fig. 7:**
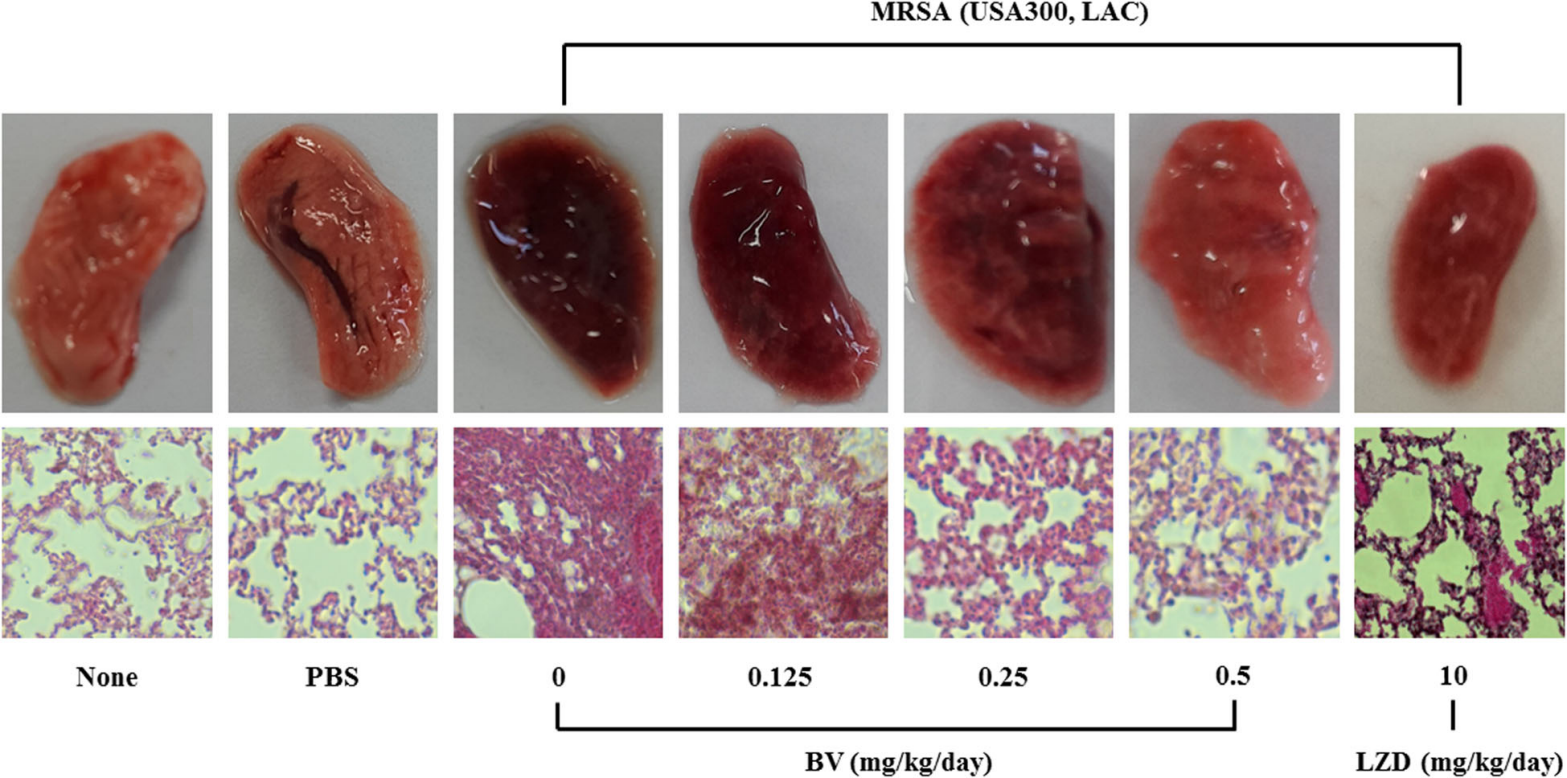
BV protection against *S. aureus* pneumonia. Gross pathological changes and histopathology of *S. aureus*-infected lung tissue from mice treated with PBS or various concentration BV (0.125, 0.25 and 0.5 mg/kg) or 10 mg/kg LZD

**Fig. 8:**
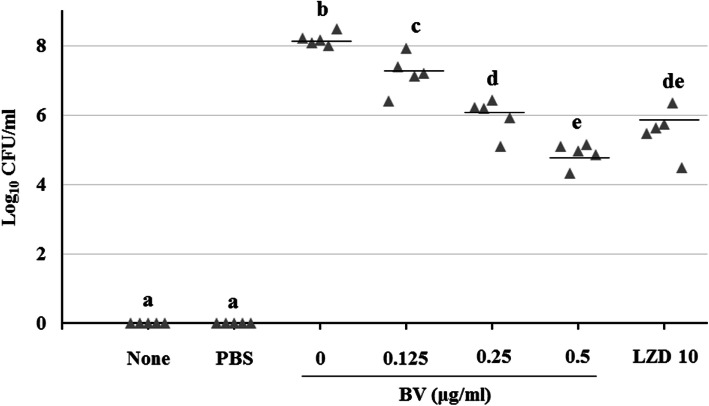
Lung bacterial clearance with various BV concentrations in the MRSA-induced pneumonia model. Bacterial clearance was evaluated at 24 h post-MRSA infection in a mouse sublethal model of MRSA pneumonia. Different letters (**a**-**e**) indicate significant differences at the *p* < 0.05 level, according to analysis of variance followed by Scheffe’s test for multiple comparisons (*p* < 0.05). Values are means ± standard errors

Mouse survival rates were determined after intraperitoneal administration of BV to pneumonia-induced model mice. As a result, it was found that 20% of the mice survived in the BV 0.125 and 0.25 mg/kg groups, and 60% survived in the BV 0.5 and LZD 10 mg/kg group (Fig. 6). Gross pathological change and H&E staining of lung tissues is shown in Fig. 7. Compared to the PBS group, more inflammation, with pulmonary edema, multifocal bacterial aggregates, and lung structure destruction, was observed in the lungs of the MRSA-infected mice. With BV treatment, no abscesses were formed, lung edema was less pronounced, and fewer inflammatory cells were observed compared to those observed without BV treatment (Fig. 7). To confirm the lung bacterial clearance, lungs of infected mice were homogenized, and then the number of bacterial cells was counted. As a result, it was shown that the number of bacterial cells decreased from 10^8^ to 10^4^ CFU/mL at the maximum. When 0.5 mg/kg BV was treated, the number of bacteria was about 10 times smaller than that of 10 mg/kg LZD treatment (Fig. 8).

### The effect of BV in inflammation-related gene expression in a lung infection model

**Fig. 9:**
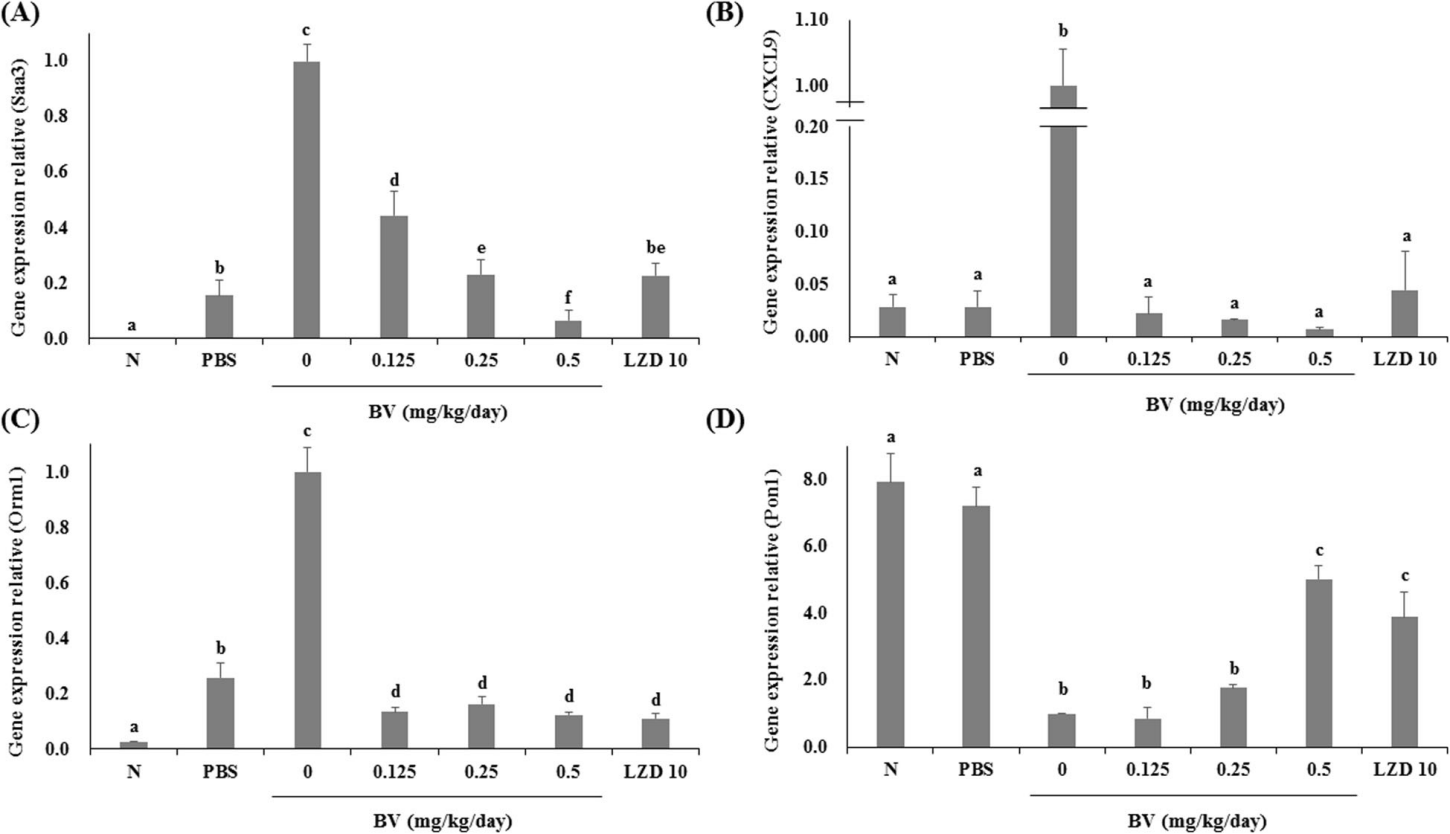
Effects of BV on mRNA expression after lung infection with MRSA. Three genes (*Saa3*, *Cxcl9*, and *Orm1*) were upregulated, and *Pon1* was downregulated upon pneumonia. Different letters (a-f) indicate significant differences at the *p* < 0.05 level, according to analysis of variance followed by Scheffe’s test for multiple comparisons (*p* < 0.05). Values are means ± standard errors

qRT–PCR was performed to evaluate the gene expression in lung tissue. Compared to their levels of expression in the PBS group, the serum amyloid A3 (Saa3), chemokine (C–X–C motif) ligand 9 (Cxcl9), and orosomucoid 1 (Orm1) genes were significantly upregulated, while the paraoxonase 1 (Pon1) gene was significantly downregulated in the lungs after MRSA infection. However, expression of Saa3, Cxcl9, and Orm1 decreased and that of Pon1 increased in the BV-treated group compared to that in the BV-free MRSA infection group (Fig. 9).

## Discussion

Pneumonia is one of the most serious infectious diseases with high morbidity and mortality. Children below the age of 5 and seniors over the age of 65 are more susceptible to developing severe pneumonia, owing to their weaker immune systems. The emergence of drug-resistant bacteria complicates the empirical treatment of pneumonia. For example, penicillin resistance has become widespread, and patients with MRSA pneumonia have a higher risk of treatment failure [20]. New antibacterial agents are urgently needed to combat this crisis, and natural products are important sources for new antibiotics. BV contains various peptides, amines, nonpeptide components, and free amino acids, which show anti-inflammatory, analgesic, and anticancer effects, such as melittin, secapin, MCD peptide, apamin, etc. [10–13]. BV has been reported to exhibit antimicrobial activity against MRSA, and its main constituent, melittin, has also been reported to show antimicrobial activity against MRSA. Therefore, we expected that BV would be effective against pneumonia caused by MRSA infection.

BV showed antimicrobial activity against MRSA and a significant bactericidal effect at relatively low concentrations (Table 3). This data showed that BV can be an effective antibiotic in the treatment of MRSA infections. The α-hemolysin is toxic substances produced by *S. aureus* and is virulence factors of infectious diseases caused by MRSA [21]. The toxin secreted by *S. aureus* causes tissue damage, promote bacterial dissemination and metastatic growth in distant organs, and enable the pathogen to evade the host innate immune response [22]. Expression of the toxin, an important factor in the pathogenicity of MRSA, was found to decrease when bacterial cells were treated with BV (Fig. 3). The secretion of TNF-α and IL-6 was decreased by BV in inflammatory response 264.7 RAW macrophages stimulated by MRSA (Fig. 4). These results suggested that BV reduces the pathogenicity by decreasing toxin production by MRSA. It was also shown that the survival rate of A549 cells treated with BV increased (Fig. 6). The *hla* gene encodes α-hemolysin. This gene is regulated by *agrA*. The agr quorum sensing, an important bacterial regulatory system that relies on the detection of extracellular autoinducers, has been shown to control the virulence of many bacterial pathogens, including regulation of α-hemolysin. In *S. aureus*, the *agr* quorum sensing is responsible for the increased expression of virulence genes, including those encoding many toxins and degradative exoenzymes, which are essential for the establishment of infection [23–26]. The results presented in Fig. 2 showed that BV decreased the *hla* and *agrA* expression in MRSA. The data suggested that BV possessed antibacterial activity and inhibited the virulence of MRSA because of the reduction of toxin production and related gene expression.

The primary aim of this study was to examine the effect of BV in a pneumonia model induced by MRSA infection. Survival rates were increased by administering BV to MRSA-induced pneumonia mice (Fig. 6). The BV-treated group showed a recovery of the lung structure destruction and a decrease of multifocal bacterial aggregates and pulmonary edema (Fig. 7.). In addition, when mice were treated with BV, the number of bacterial cells present in the lungs was reduced (Fig. 8). The α-hemolysin, a pore-forming cytolytic toxin, has been shown to be a key virulence determinant in *S. aureus*-induced pneumonia mouse models [27]. By inhibiting the production and activity of α-hemolysin in MRSA, it is possible to alleviate the pneumonia caused by the infection. BV reduced the expression levels of α-hemolysin and the *hla* gene, which encodes α-hemolysin, in MRSA. This result suggested that BV protects lung tissue from the pathogenicity of MRSA.

We also confirmed the expression of inflammatory genes in pneumonia. Serum amyloid A-3 (SAA3) is an acute phase response protein whose transcription is strongly induced by endotoxin-induced lung inflammation [28]. Chemokine ligand 9 (CXCL9) is a small cytokine belonging to the CXC chemokine family, which is induced by inflammatory responses and is also known to be induced by pulmonary inflammation [29]. Orm1 is a gene encoding the orosomucoid 1 (ORM1) protein, which is classified as an acute phase protein and is increased by an acute inflammatory reaction [30]. Pon1 is a gene encoding paraoxonase 1 (PON1). Paraoxonases have been found to be show a number of biological functions such as anti-inflammatory, antioxidative, antimicrobial, and organophosphate-hydrolyzing activities. Sepsis caused by MRSA may contribute to an oxidative environment, which may result in increased binding of free radicals to PON1, leading to a reduction in PON1 activity in the circulation [31]. Our results showed that the expression of Saa3, Cxcl9, and Orm1 in the BV-treated group was reduced compared to that in the untreated group. However, the expression level of Pon1 in the BV-treated group increased compared to that in the untreated group (Fig. 9). These results suggested that the inflammatory reaction was alleviated in pneumonia-induced lung tissue because of decrease of virulence factor by BV.

## Conclusion

In summary, BV showed antibacterial activity against MRSA and inhibited toxin production. These activities of BV reduce the virulence of the bacterium and the number of bacterial cells present in lung tissue, thereby alleviating the symptoms of pneumonia in mice. This study suggested that BV may be a candidate substance for the treatment of pneumonia caused by MRSA infection. We plan to study the mechanism of this activity and various physiological activities of BV in the future.

## Supplementary information

**Additional file 1.**

## Data Availability

The datasets used and/or analysed during the current study available from the corresponding author on reasonable request.

## Abbreviation

BV: Bee venom
MRSA: Methicillin-resistant *Staphylococcus aureus*
hla: α-hemolysin
LZD: Linezolid
MIC: Minimum inhibitory concentration
MHB: Mueller–Hinton broth
TSB: Tryptic soy broth
LDH: Lactate dehydrogenase
