# Correction to: The antibacterial activity and toxin production control of bee venom in mouse MRSA pneumonia model

**DOI:** 10.1186/s12906-020-03108-x

**Published:** 2020-10-14

**Authors:** Dong-Yeul Kwon, Ryong Kong, Young-Seob Lee, Dam-Hee Kang, Shu Wang, Qianqian Li, Ok-Hwa Kang

**Affiliations:** 1 Department of Oriental Pharmacy, College of Pharmacy and Wonkwang-Oriental Medicines Research Institute, Wonkwang University, Iksan, Jeonbuk 54538 Republic of Korea; 2 Department of Herbal Crop Research, National Institute of Horticultural & Herbal Science, RDA, 92 Bisanro, Eumsung, Chungbuk 27709 Republic of Korea

**Correction to: BMC Complement Med Ther (2020) 20:238**

**https://doi.org/10.1186/s12906-020-02991-8**

Following publication of the original article [1], the authors identified an error in order of names of authors. The correct order of authors should be as given below.

Dong-Yeul Kwon^1^, Ryong Kong^1^, Young Seob Lee^2^, Dam-Hee Kang^1^, Shu Wang^1^, Qianqian Li^1^, and Ok-Hwa Kang^1*^
