# Supplementary figures and images for: The antibacterial activity and toxin production control of bee venom in mouse MRSA pneumonia model
Source: BMC Complement Med Ther. 2020 Jul 27;20:238. doi: 10.1186/s12906-020-02991-8 (PMC7385961; doi:10.1186/s12906-020-02991-8)

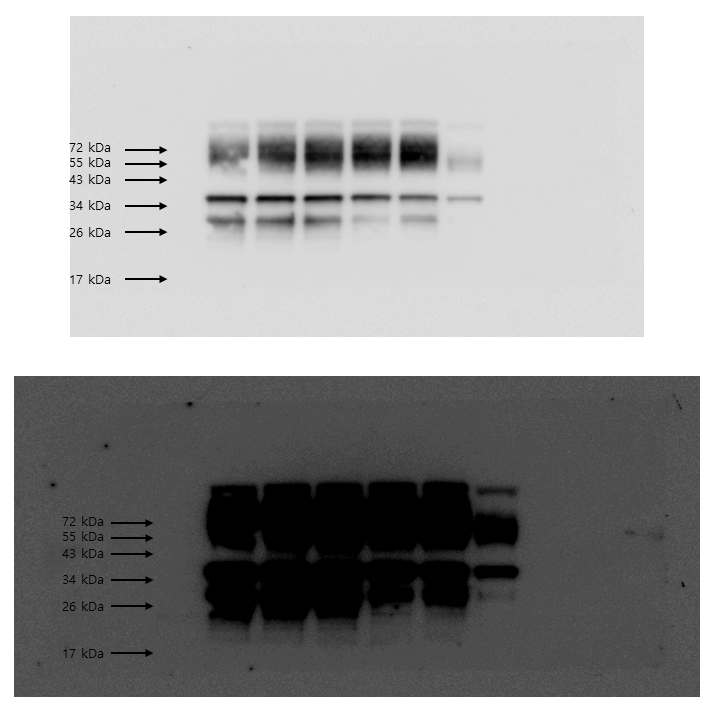

Supplement: Supplementary file 1 — Additional file 1. [file 12906_2020_2991_MOESM1_ESM.tif]
